# Odorant-Binding Proteins Contribute to the Defense of the Red Flour Beetle, *Tribolium castaneum*, Against Essential Oil of *Artemisia vulgaris*

**DOI:** 10.3389/fphys.2020.00819

**Published:** 2020-08-31

**Authors:** Yuan-chen Zhang, Shan-shan Gao, Shuang Xue, Kun-peng Zhang, Jing-shun Wang, Bin Li

**Affiliations:** ^1^College of Biology and Food Engineering, Anyang Institute of Technology, Anyang, China; ^2^College of Life Sciences, Nanjing Normal University, Nanjing, China

**Keywords:** *Tribolium castaneum*, essential oil, odorant-binding proteins, toxic substance, RNA interference

## Abstract

The function of odorant-binding proteins (OBPs) in insect chemodetection has been extensively studied. However, the role of OBPs in the defense of insects against exogenous toxic substances remains elusive. The red flour beetle, *Tribolium castaneum*, a major pest of stored grains, causes serious economic losses for the agricultural grain and food processing industries. Here, biochemical analysis showed that essential oil (EO) from *Artemisia vulgaris*, a traditional Chinese medicine, has a strong contact killing effect against larvae of the red flour beetle. Furthermore, one OBP gene, *TcOBPC11*, was significantly induced after exposure to EO. RNA interference (RNAi) against *TcOBPC11* led to higher mortality compared with the controls after EO treatment, suggesting that this OBP gene is associated with defense of the beetle against EO and leads to a decrease in sensitivity to the EO. Tissue expression profiling showed that expression of *TcOBPC11* was higher in the fat body, Malpighian tubule, and hemolymph than in other larval tissues, and was mainly expressed in epidermis, fat body, and antennae from the early adult. The developmental expression profile revealed that expression of *TcOBPC11* was higher in late larval stages and adult stages than in other developmental stages. These data indicate that *TcOBPC11* may be involved in sequestration of exogenous toxicants in the larvae of *T. castaneum*. Our results provide a theoretical basis for the degradation mechanism of exogenous toxicants and identify potential novel targets for controlling the beetle.

## Introduction

Insects have evolved a sensitive olfactory system to detect diverse odor molecules in their habitation environment. Using the olfactory system, insects are able to carry out various physiological and reproductive activities (Pelosi et al., 2018; Yan et al., 2020). Odor chemicals, bound with some proteins in the sensillum lymph, are transported to odorant receptors across the sensillar lymph, and ultimately activate a series of signaling pathways (Pelosi et al., 2006; Leal, 2013). Proteins that bind odor chemicals include odorant binding proteins (OBPs) and chemosensory proteins (CSPs). Interactions between odorants and OBPs likely trigger the signal transduction process of odorant recognition in insects (Leal et al., 2005; Smith, 2007; Rong et al., 2015). Since the first insect OBP was discovered in the female antennae of *Antheraea polyphemus* (Vogt and Riddiford, 1981), numerous OBPs have been identified in a diverse range of insects using protein ligand-binding and nucleotide sequencing methods (He et al., 2019). For example, 52 OBPs were identified in *Drosophila melanogaster* (Menuz et al., 2014), 44 in *Bombyx mori* (Gong et al., 2009), and 21 in *Apis mellifera* (Foret and Maleszka, 2006). Although the function of OBPs in olfaction has been extensively studied, the role of these proteins in non-olfactory processes is poorly understood.

Insect OBPs are highly soluble, globular proteins (15–20 kDa) that are secreted at high concentrations in the sensillar lymph (Pelosi et al., 2018; Sun et al., 2018). Although OBPs are highly divergent between insects, they contain conserved cysteine residues that are bonded by interlocked disulfide bridges (Leal et al., 1999; Scaloni et al., 1999). OBP genes are expressed at higher levels in adult antennae than in other apparatus, suggesting their participation in odorant identification by adult insects (Chang et al., 2017; Huang et al., 2018; Zhang et al., 2020). The functions of OBPs have been verified by means of *in vitro* binding experiments in a diverse range of insects such as *D. melanogaster* (Larter et al., 2016), *B. mori* (Zhou et al., 2009), *Aedes aegypti* (Kim et al., 2017), and *Periplaneta* americana (He et al., 2017). Exposure to exogenous toxicants resulted in significant elevation in expression of *OBPs* in insects, coupled with a gradual increase in resistance to the toxicants (Bautista et al., 2015; Li Z. Q. et al., 2015; Li et al., 2016; Liu et al., 2017; Xiong et al., 2019). These studies suggested that OBPs were involved in exogenous toxicant resistance in insects. However, the roles of OBPs in the insect defense against exogenous pesticides remain elusive.

*Tribolium castaneum* (Herbst) (Coleoptera: Tenebrionidae), generally known as the red flour beetle, can seriously damage stored agricultural grains and their processed products (Islam, 2017). Deterioration in quantity and quality of the stored grain due to this insect pest causes an annual economic loss of billions of dollars (Li et al., 2011). Control of this pest currently depends on fumigation with phosphine due to restrictions on the use of other insecticidal agents against this pest (Bulter and Rodriguez, 1996). However, misuse of phosphine fumigation has led to strong resistance (Kumar et al., 2011; Caballero-Gallardo et al., 2012). Some studies showed that essential oils of plants can be used as a biological agent to control *T. castaneum* instead of pesticides (Ebadollahi and Jalali Sendi, 2015; Upadhyay et al., 2018). However, many plants contain thujone, which is harmful to human health (Pelkonen et al., 2013). *Artemisia vulgaris*, a traditional Chinese plant, does not contain thujone (Jiang et al., 2019) and the essential oil of this plant could therefore potentially be used to control *T. castaneum*. In the present study, essential oil from *A. vulgaris* (EO-AV) was extracted and its contact killing effect on *T. castaneum* larvae was assessed by the drip method. Essential oils of plants, as an exogenous pesticide, generally induce defense reactions in insects (Wei et al., 2019). RNA-Seq analysis revealed that one *OBP* gene (GenBank accession number NC_007425) in the larva of *T. castaneum* was highly expressed in the presence of EO-AV (Log_2_Ratio of FPKM of *OBPC11* was 4.02). Based on these results, the function of the OBP in the defense of *T. castaneum* larvae against EO-AV was further dissected.

## Materials and Methods

### Insect Rearing

*T*. *castaneum* were fed in an artificial climate box with a temperature of 30°C and humidity of 40%. The food was made of flour and yeast (19:1) under standard conditions (Xiong et al., 2019).

### Preparation of Essential Oil of *Artemisia vulgaris*

*A. vulgaris* was collected in December 2017 at Mowan Village, Tangyin City, Henan Province, China (l35.922862°N, 114.480455°E). The collection site has a warm temperate continental monsoon climate, with adequate light, an average annual temperature of 14°C, average annual rainfall of 580 mm, and is located at an altitude of approximately 63 m above sea level. Fresh leaves and stems of *A. vulgaris* were washed with ddH_2_O then placed inside a dark box at 37°C to dry for 1 week. Dried leaves were shattered using a Swing Medicinal material grinder (Baijie Industrial Co., Ltd., Shanghai, China). The powder was filtered using 30 mesh sieves. Filtered powders (200 g) were loaded into a 5-L extraction tank. When the vacuum pressure in the tank reached 100 kPa, a subcritical solvent (dimethylmethane, butane, dimethyl ether, and tetrafluoroethane mix) was injected into the tank. Extraction began at 35°C, with a liquid-solid ratio of 15: 1. Each extraction was 30 min, with three repeats during the whole extraction process. At the end of the process, the liquid solvent reached the separation tank and, after the solvent was removed by evaporation, the EO-AV was collected. The extract was diluted with acetone to obtain six concentrations: 0, 2.5, 5, 10, 15, and 20%.

### Contact Killing Effect of Essential Oil Against *T*. *castaneum*

The contact killing effect of EO-AV against late larvae (20 days old) of *T*. *castaneum* was measured according to the drip method of Lu et al. (2012). Briefly, 30 synchronous individuals in each group were loaded into 1.5-mL EP tubes and exposed to 100 μL EO-AV or acetone. After soaking for 1 min, the treated larvae were placed on filter paper and allowed to air dry for 2 min. Each group was then transferred to an 8-mL glass vial and maintained under standard conditions described in section “Insect Rearing.” Survival of individual larvae in the different treatment groups was recorded from 12 to 72 h after EO-AV exposure. Beetles were considered dead if they were unable to move and failed to respond when disturbed with a tweezer or brush. Each bioassay was replicated five times.

### Identification and Cloning of OBP Genes in *T*. *castaneum*

EO-AV had a strong contact killing effect on late larvae of *T. castaneum*. Therefore, 36 h after the treatment with 5% essential oil (TG) or 0% essential oil (CK), the late larvae were collected to extract RNA for RNA-Seq (Gao et al., 2020). Expression of the gene *OBPC11* was significantly upregulated in the TG vs CK samples; consequent experiments in this study focused on the role and function of this gene. Primers were designed against OBPC11 from *T. castaneum* (*TcOBPC11*) to obtain the full-length cDNA sequence of the open reading frame (ORF) (Table 1). Total RNA was isolated from the late larvae using Trizol reagent (Vazyme Biotech, Nanjing, China), and 750 ng total RNA was used to synthesize cDNA templates using HiScript Reverse Transcriptase (Vazyme Biotech). These cDNA templates were used to clone the OBP gene by PCR in a 50 μL reaction comprising 2 μL cDNA, 25 μL 2 × Primer STAR Mix, 1 μL forward and reverse primers (10 μM), and 21 μL sterile water. The PCR amplification procedure was 95°C for 3 min, then 35 cycles of 95°C for 20 s, 55°C for 15 s, and 72°C for 30 s, followed by a final extension at 72°C for 10 min. The PCR product was detected using 1.2% agarose gel and the expected product was purified by FastPure Gel DNA Extraction Mini Kit (Vazyme Biotech), cloned into Blunt-Zero Vector via Blunt-Zero Cloning Kit (Vazyme Biotech), and then transformed into competent cells of *Escherichia coli*. Positive clones were identified by blue-white screening and the resulting vector was extracted and sequenced (Springen). In addition, 30 late larvae were collected to extract RNA for qPCR after treatment with 5 or 0% essential oil for 12, 24, 36, 48, 60, and 72 h.

**TABLE 1:** Primers used for this research.

**Primer**	**Primer sequence (5′→3′)**	**Product length (bp)**	**Purpose**
OBP-1F	ATGAAAATTGTTTTGTGCCTTCT	402	Amplification of ORF of *OBPC11*
OBP-1R	TCACTCATCGACCGAATTTTT		
OBP-2F	ATTGTTTTGTGCCTTCTTGCC	293	qPCR of *OBPC11* gene
OBP-2R	GTCCCTTCAGCTTCATTGCTC		
rps3-F	ATGAAAATTGTTTTGTGCCTTCT	260	qPCR of reference gene
rps3-R	TCACTCATCGACCGAATTTTT		
dsOBP-F	TAATACGACTCACTATAGGGAGA TGATGAACGATGCTGGAGA	171	RNAi of *OBPC11* gene
dsOBP-R	TAATACGACTCACTATAGGGAAT TTTTGTTGCGGATGAGACA		
dsGFP-F	TAATACGACTCACTATAGGG CGATGCCACCT	256	RNAi of *GFP* gene
dsGFP-Rs	TAATACGACTCACTATAGGG TGTCGCCCTCG		

*F represents forward primers; R represents reverse primers. The underlined letters are the T7 promoters for dsRNA synthesis.*

### Expression Profiling of the Gene *TcOBPC11*

Pooled samples of male and female *T*. *castaneum* in different developmental stages, including early eggs (1 day old), late eggs (3 days old), early larvae (1 day old), late larvae (20 days old), early pupae (1 day old), late pupae (5 days old), early adults (1 day old), and late adults (10 days old), were collected, snap-frozen in liquid nitrogen, and immediately stored at −80°C to extract RNA for qPCR. Pooled samples of different tissues from the late larvae (whole larva, central nervous system, epidermis, fat body, Malpighian tubule, gut, and hemolymph) and from early adults (whole adult, central nervous system, epidermis, fat body, Malpighian tubule, gut, antennae, testis, and ovary) were dissected and collected in RNA-free centrifuge tubes to extract RNA for qPCR. Three biological replicates for each developmental stage and tissues were conducted.

### Quantitative RT-PCR Analyses

Firstly, 750 ng total RNA from each developmental stage and the different tissues were treated with 4 × gDNA wiper Mix to avoid the effects of genomic DNA on qPCR. Total RNA was then used as a template to synthesize single-stranded cDNAs using HiScript Q RT SuperMix for qPCR with Random primers and Oligo dT primer mix (Vazyme Biotech) in 25 μL reactions. The specific primers designed to quantify *TcOBPC11* expression were OBP-2F and OBP-2R. The gene *ribosomal protein S3* (rps3), which has a high degree of stability, was selected as the reference gene (Toutges et al., 2010; Horn and Panfilio, 2016; Xiong et al., 2019) and the primers were rps3-F and rps3-R. Primers were designed using Primer Premier 5.0 and sequences are shown in Table 1. Primers of the target gene and the reference gene have similar amplification efficiencies. A 10-μL reaction system containing 0.25 μL forward and reverse primers (10 μM), 5 μL 2 × AceQ Universal SYBR qPCR Master Mix, 3.5 μL ddH_2_O water, and 1 μL cDNA was executed in an ABI Q6 (Applied Biosystems, Foster City, CA, United States) with the parameters of 95°C for 10 min, 40 cycles of 95°C for 15 s, and 60°C for 60 s, followed by 95°C for 15 s, 60°C for 60 s, and 95°C for 15 s. A melting curve of the amplification products was generated at the end of each reaction to confirm that only one PCR product was amplified. For each treatment, three technical repeats and three to seven biological replicates were executed.

### Double-Stranded RNA (dsRNA) Synthesis and Injection

To synthesize the dsRNAs, primers for *TcOBPC11* and *GFP* were designed using Primer Premier 5.0, adding a T7 polymerase recognition promoter (Table 1). The target of the dsRNAs is located from 214 to 382 bp in the coding region of *TcOBPC11*. The PCR comprised 0.4 μL forward and reverse primers (10 μM), 10 μL 2 × Primer STAR Mix, 7.2 μL ddH_2_O water, and 2 μL plasmid DNA with ORF cDNA of *OBPC11*. PCR procedures were carried out at 95°C for 5 min followed by 30 cycles of 95°C for 45 s, 60°C for 45 s, and 72°C for 15 s, with a final extension at 72°C for 10 min. PCR products were purified and served as templates to synthesize the dsRNAs using a TranscriptAi T7 High Yield Transcription Kit (Fermentas, Vilnius, Lithuania). The resulting dsRNA (200 ng) in a volume 150 nL solution was injected into the body cavity of each late larva of *T*. *castaneum* by InjectMan 4 (Eppendorf, Hamburg, Germany). Injection of equal volumes of 200 ng dsGFP and water served as the negative and blank controls, respectively. Three biological repetitions with independent injections were performed and each repetition injected 30 larvae. Late larvae injected with dsRNA were normally reared. Survival of the larvae was recorded after the fifth day. Ten random larvae were collected on the first, third, and fifth days to extract total RNA for qPCR to detect the silencing efficacy of the targeted gene. To verify the effect of non-target genes, expression of four non-target genes (*TcOBPC01*, *TcOBPC02*, *TcOBPC04*, and *TcOBPC10*) in *T*. *castaneum*, which share a high degree of similarity with *TcOBPC11*, were also examined; primer details for these genes are listed in Supplementary Table S1. Late larvae on the fifth day after water, dsGFP, dsOBPC11 injections, and non-injections were exposed to 5% EO-AV for 1 min. Insects were then transferred to the standard conditions, reared for 36 h, and their mortality was measured. Each treatment was repeated three to seven times.

### Data Analysis

Gene expression was calculated according to the 2^–△△*Ct*^ method (Livak and Schmittgen, 2001). Significant differences in gene expression levels between larvae of *T*. *castaneum* treated with 0.5% essential oil and 0% essential oil were compared using a Student’s *t*-test at the 0.05 level. Expression levels of *TcOBPC11* among different developmental stages and RNAi treatments were analyzed by Fisher’s LSD test. After logarithmic transformation of levels of expression of *TcOBPC11* in different tissues, significant differences were analyzed by Fisher’s LSD test. Statistical significance of the mortality or survival rate were analyzed by Kruskal-Wallis H test, except in the RNAi experiment, which was analyzed by Fisher’s LSD test. Interaction effects of the concentration of EO-AV and time after exposure to EO-AV on the mortality rate of *T. castaneum* larvae were investigated with a non-parametric two-way ANOVA (Scheirer–Ray–Hare test) to assess significant differences at the 0.05 level. All statistical analyses were performed with SPSS software, version 14.0.

## Results

### Contact Killing Effect of EO-AV Against Larvae of *T*. *castaneum*

**FIGURE 1:**
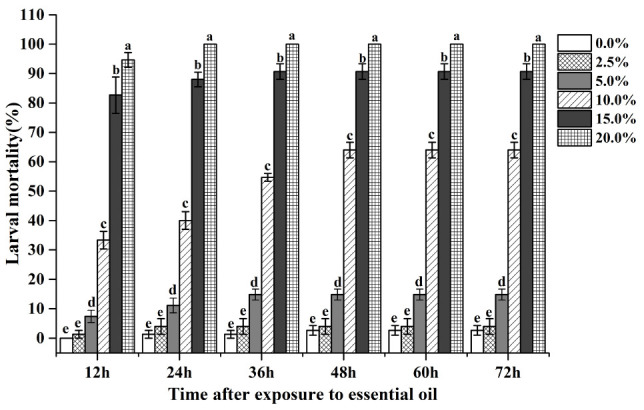
Mortality rates of larvae at 12, 24, 36, 48, 60, and 72 h after exposure to different concentrations of the essential oil of *A. vulgaris*. Different letters above the bars (mean ± SE, *n* = 5) indicate significant differences (*P* < 0.05) among the six concentrations at the same time.

There were significant differences in the mortality of larvae of *T*. *castaneum* exposed to different concentrations of EO-AV when reared for the same length of time (Figure 1 and Table 2). Larva mortality increased significantly as the concentration of EO-AV increased from 0 to 20% (12 h: *x^2^* = 27.217, *df* = 5, *P* < 0.001; 24 h: *x^2^* = 27.293, *df* = 5, *P* < 0.001; 36 h: *x^2^* = 27.872, *df* = 5, *P* < 0.001; 48 h: *x^2^* = 27.590, *df* = 5, *P* < 0.001; 60 h: *x^2^* = 27.620, *df* = 5, *P* < 0.001; 72 h: *x^2^* = 27.620, *df* = 5, *P* < 0.001). There were significant differences in the mortality of larvae at different time points after exposure to the same concentration of EO-AV (Figure 1 and Table 2). Mortality of *T*. *castaneum* larvae significantly increased following the extension of rearing time after exposure to EO-AV at concentrations of 10, 15, and 20% (10%: *x^2^* = 24.366, *df* = 5, *P* < 0.001; 15%: *x^2^* = 20.875, *df* = 5, *P* < 0.001; 20%: *x^2^* = 16.071, *df* = 5, *P* = 0.007), but there was no significant effect at concentrations of 0, 2.5, and 5% (0%: *x^2^* = 2.231, *df* = 5, *P* = 0.816; 2.5%: *x^2^* = 3.295, *df* = 5, *P* = 0.655; 5%: *x^2^* = 6.364, *df* = 5, *P* = 0.272). In a non-parametric two-way ANOVA, mortality rates of the red flour beetle were affected by concentration and rearing time after exposure to EO-AV, but the interaction was not significant (Table 2).

**TABLE 2:** Effect of concentration and rearing time on mortality rate after exposure to EO-AV^*a*^.

**Source of variation**	***df***	**Mean square**	***F***	***p***
Concentration	5	86,970.783	586.682	<0.001
Time	5	2,165.170	14.606	<0.001
Concentration × time	25	162.037	1.093	0.358

*^*a*^Determined by non-parametric two-way ANOVA (Scheirer–Ray–Hare test).*

### Identification and Characterization of the Gene *TcOBPC11*

Transcriptome analysis revealed that 39 chemosensory-related genes had significant differences in expression between the TG group and the CK group (Gao et al., 2020). These genes included six OBPs, five CSPs, six odorant receptors, and three gene families encoding odorant-degrading enzymes: 14 cytochrome P450s, three esterases, and five glutathione S-transferases (Supplementary Table S2). Among these genes, expression of *TcOBPC11* was highest in the TG group compared with the CK group (Gao et al., 2020). To appraise the underlying function of *TcOBPC11* in the tolerance of *T. castaneum* to EO-AV, cDNA of *TcOBPC11* with an ORF of 402 bp, encoding a protein of 134 amino acids, was cloned (GenBank accession no. XM_962706). A phylogenetic tree of the TcOBPC11 protein with homologous proteins of other insects was reconstructed (Supplementary Figure S1). The similarity of *TcOBPC11* with homologous genes from other insects was very low. TcOBPC11 was most related to an OBP in *Asbolus verrucosus* and *Tenebrio molitor* (Supplementary Figure S1). The amino acid identities of *TcOBPC11* with OBP of *A. verrucosus* and *T. molitor* were 40.04 and 35.50%, respectively.

### EO-AV Induced Upregulated Expression of *TcOBPC11*

**FIGURE 2:**
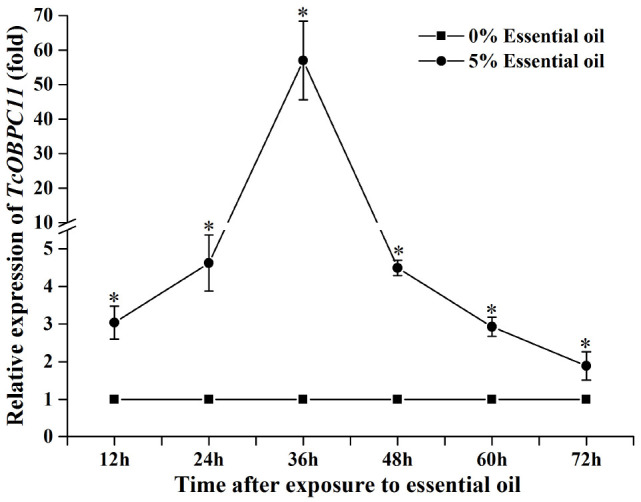
Relative expression of *TcOBPC11* at 12, 24, 36, 48, 60, and 72 h after exposure to 5 and 0% EO-AV. Asterisks above the bars (mean ± SE, *n* = 3) indicate a significant difference between the treatments of 5 and 0% EO-AV (*P* < 0.05) at the same time point.

Expression of the gene *TcOBPC11* was significantly higher in TG than CK samples using qPCR methods (36 h: *t* = 4.917, *df* = 3, *P* = 0.016) (Figure 2); this was consistent with the transcriptome analysis results of Gao et al. (2020). *TcOBPC11* had significantly higher expression at each time point after exposure to 5% EO-AV compared with 0% EO-AV (Figure 2). There were significant differences in expression among 12, 24, 36, 48, 60, and 72 h at a concentration of 5% EO-AV (*F*_5, 23_ = 29.300, *P* < 0.001). Meanwhile, after exposure to 5% EO-AV, expression of *TcOBPC11* rapidly increased from 24 h, peaked at 36 h, and then decreased gradually (Figure 2). This indicated that EO-AV induced expression of the gene *TcOBPC11*.

### Developmental Stage and Tissue-Specific Expression Profiles of *TcOBPC11*

**FIGURE 3:**
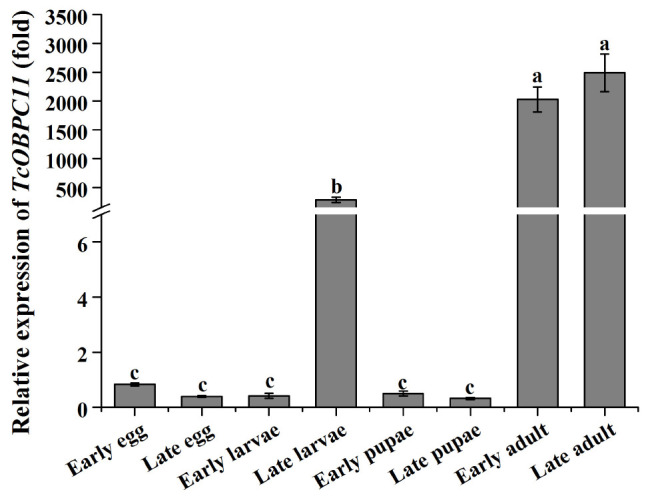
*TcOBPC11* expression patterns in eight key developmental stages from *T. castaneum*, including early eggs (1 day old), late eggs (3 days old), early larvae (1 day old), late larvae (20 days old), early pupae (1 day old), late pupae (5 days old), early adults (1 day old), and late adults (10 days old). Transcript levels of the target transcripts in early eggs served as the calibrator for the developmental expression profiling. Vertical bars indicate standard errors of the mean (*n* = 3∼5) and different letters on the bars indicate that the means are significantly different among the different developmental stages at the *P* < 0.05 level.

qRT-PCR revealed that *TcOBPC11* was expressed throughout all stages of development of *T*. *castaneum* and there were significant differences in expression of *TcOBPC11* among developmental stages (*F*_7, 24_ = 1,660.983, *P* < 0.001) (Figure 3). *TcOBPc11* was highly expressed at the late larvae and adult stage, and expression at these stages was significantly greater than at other developmental stages (Figure 3).

**FIGURE 4:**
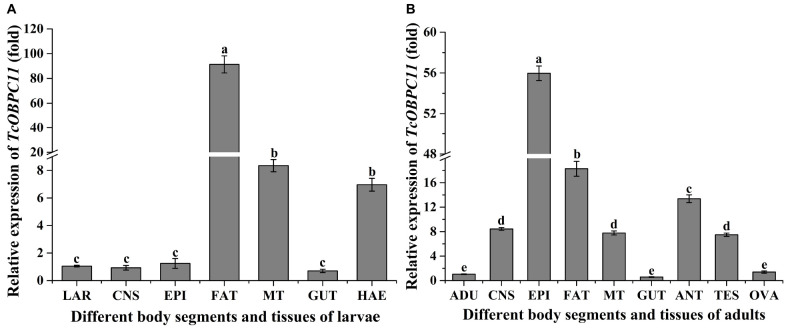
*TcOBPC11* expression patterns in different tissues of *T. castaneum* larvae **(A)**, including whole larvae (LAR), central nervous system (CNS), epidermis (EPI), fat body (FAT), Malpighian tubule (MT), gut (GUT), and hemolymph (HAE), and in different tissues of *T. castaneum* adults **(B)**, including whole adults (ADU), central nervous system (CNS), epidermis (EPI), fat body (FAT), Malpighian tubule (MT), gut (GUT), antennae (ANT), testis (TES), and ovary (OVA). The transcripts level of *TcOBPc11* in the whole larvae or whole adults were used as the calibrator for the tissue-specific expression profiling. Vertical bars indicate standard errors of the mean (*n* = 3) and different letters on the bars indicate that the means are significantly different among the different tissues at the *P* < 0.05 level.

Expression of *TcOBPC11* was further surveyed in various tissues from late larvae and early adults. Relative transcript levels of *TcOBPC11* in different tissues from the larvae of *T*. *castaneum* showed that *TcOBPC11* was mainly expressed in the fat body, Malpighian tubule, and hemolymph, and at much lower levels in other tissues (*F*_6, 14_ = 318.323, *P* < 0.001) (Figure 4A). Expression of *TcOBPC11* in larvae was highest in the fat body, closely followed by the Malpighian tubule and hemolymph, and then other apparatus (Figure 4A). Relative transcript levels of *TcOBPC11* in different tissues from adult *T*. *castaneum* showed that *TcOBPC11* was mainly expressed in the epidermis, fat body, and antennae, and at much lower levels in other tissues (*F*_6, 14_ = 318.323, *P* < 0.001) (Figure 4B). Expression of *TcOBPC11* in adults was highest in the epidermis, closely followed by the fat body and antennae, and then other apparatus (Figure 4B). *TcOBPC11* was highly expressed in olfactory and non-olfactory tissues, suggesting that *TcOBPC11* has multiple physiological functions in *T*. *castaneum*.

### Functional Analysis of *TcOBPc11* by RNAi Methods

**FIGURE 5:**
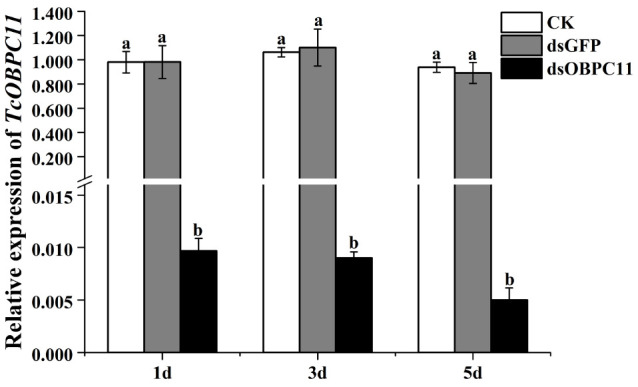
Relative expression of *TcOBPC11* mRNA in *T*. *castaneum* on days 1, 3, and 5 after injection of dsOBPC11. Control larvae were injected with the same amount of water. Error bars represent the standard error of three biological replicates. Different lowercase letters above the bars indicate significant differences between treatments at the *P* < 0.05 level.

To further determine the effects of *TcOBPC11* on insect resistance to EO-AV, RNAi was used to silence the gene *TcOBPC11*. RNAi targeting of *TcOBPC11* in late larvae distinctly lowered its expression level but did not change the transcript level of non-target genes (Supplementary Figure S2). This indicates that RNAi of *TcOBPC11* was absent of off-target effects. Relative expression levels of *TcOBPC11* mRNA significantly decreased after injection of dsOBPC11 (day 1: *F*_2, 6_ = 36.116, *P* < 0.001; day 3: *F*_2, 6_ = 46.395, *P* < 0.001; day 5: *F*_2, 6_ = 88.310, *P* < 0.001) (Figure 5). At day 5 after injection of water, dsOBPC11, or dsGFP, survival rates of *T. castaneum* larvae were 96.33 ± 3.67%, 98.38 ± 0.83%, and 93.92 ± 3.25%, respectively, and there were no significant differences between the treatments (*F*_2, 6_ = 0.605, *P* = 0.576).

**FIGURE 6:**
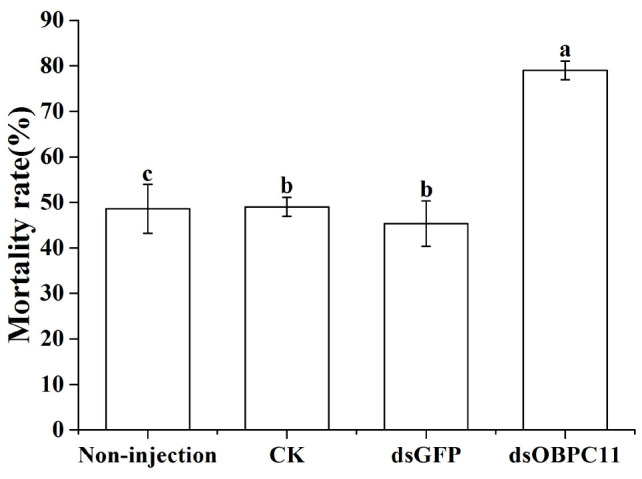
Mortality rate of *T. castaneum* larvae exposed to essential oil after injection of dsOBPC11. The bioassays were completed for late larvae on the fifth day following injection of dsRNA by dip of essential oil and the mortality rate of the larvae were assessed 36 h after dip treatments. Control larvae were injected with the same amount of water or dsGFP, or were not injected (Non-injection). The error bars represent the standard errors of three to seven biological replicates. Different lowercase letters above the bars indicate significant differences between treatments at the *P* < 0.05 level.

Dip bioassays of EO-AV were performed on the fifth day after late larvae were injected or not with dsRNA. The cumulative mortality rate of *T. castaneum* larvae increased in the non-injection, water, dsGFP, and dsOBPC11 groups because of the exposure to essential oil. However, the mortality rate of the larvae injected with dsOBPC11 significantly increased compared with the water and dsGFP groups (*F*_3, 12_ = 6.707, *P* = 0.007) (Figure 6). This supported the conclusion that the enhanced mortality rate of *T. castaneum* larvae detected after exposure to essential oil was primarily because *TcOBPC11* genes were silenced. Obvious correlation effects between the reduction in *TcOBPC11* transcript level and the enhanced mortality rate after exposure to essential oil suggest that *TcOBPC11* plays a significant role in the defense of *T. castaneum* larvae against EO-AV.

## Discussion

*Artemisia* plants have been widely applied in the field of medicine as treatments for multiple diseases (Kumar et al., 2019). The safety of these plants for human use has led to interest in the potential insecticidal activity of essential oils from the plants (Pandey and Singh, 2017). Essential oil of *Artemisia* displayed contact, fumigation, and repellent toxicity to adult or larvae of *T. castaneum* (Abou-Taleb et al., 2015; Hu et al., 2019). In this study, contact toxicity of EO-AV was investigated against the larvae of red flour beetles. Mortality of the larvae was more than 90% after 24 h of exposure to 20% EO-AV, indicating that EO-AV had a very strong toxic effect against the beetle larvae. Eucalyptol (28.07%) is the most abundant compound in *A. vulgaris* (Jiang et al., 2019). Eucalyptol has activity against various insects (Omara et al., 2018) and has been widely used as an insecticide to control crop pests (Xu, 2019). EO-AV contains other complex compounds in addition to eucalyptol. Some of these compounds, such as α-terpineol (Pandey and Singh, 2017; Hu et al., 2019) and β-pinene (Liu et al., 2010; Benelli et al., 2018), can result in high mortality of insects. The synergistic relationship of complex compounds may be responsible for the high mortality of the larvae of *T. castaneum* after treatment with EO-AV.

Several studies have shown that OBPs are transcribed in both the larval phases and the adult phases of insects (Zhou et al., 2015; Xue et al., 2016; Zhang et al., 2018). In this study, *TcOBPC11* was expressed throughout all development phases from egg to adult, suggesting that *TcOBPC11* had roles in a variety of physiological processes. Recent studies have found that expression of OBPs was prominently upregulated in the adult phase of many insects, for example the gene *CcapOBP83a*-2 from *Bactrocera capitata* (Siciliano et al., 2014), *BdOBP56d*, *BdOBP99a*, *BdOBP99c*, and *BdOBP19* from *B. dorsalis* (Zhang et al., 2018), and *OBP1*, *OBP3*, *OBP8*, *OBP11*, and *OBP24* from *Chilo suppressalis* (Yang et al., 2016). This has led to speculation that OBP genes were related to the detection of heterosexual behavior and oviposition in adult insects (Zhang et al., 2018). Our results reveal that *TcOBPC11* was highly expressed in antennae at the adult stage of *T. castaneum*. We presume this is most likely related to mating behavior and not oviposition because *T. castaneum* inhabits wheat flour and therefore does not need to select a specific location to lay eggs. *TcOBPC11* was also expressed at a high level in late larval stages, suggesting that it likely participates in the development and growth of insects. Moreover, this characteristic of high expression in late larvae was observed for the OBP gene *TcOBPC11* and is presumed to provide protective functions for insects against exogenous toxic molecules (Xiong et al., 2019). Hence, *TcOBPC11* may have a similar defense function against toxic substances in larval stages. Further studies are required to confirm the defense mechanism of *TcOBPC11* against toxic substances in larval stages.

Consistent with previous studies in which OBPs were expressed in non-olfactory tissues (He et al., 2011, 2019), *TcOBPC11* was predominantly expressed in the fat body, Malpighian tubule, and hemolymph in the late larvae. Abundant expression of *OBPs* has been observed in these tissues from other insects. In the fat body from the 5th larvae, *BmorOBP27* and *BmorOBP44* in *B. mori* showed a high level of expression (Gong et al., 2009), and two *OBPs* in *Dendrolimus punctatus* were highly expressed (Zhang et al., 2017). Many OBP genes were distributed in Malpighian tubules, such as various *OBPs* in *B. dorsalis* (Chen X. F. et al., 2019), *TcOBPC01* in *T. castaneum* (Xiong et al., 2019), and one *OBP* in *Manduca sexta* (Vogt et al., 2015). Insect hemolymph harbors OBP proteins (Kim et al., 2017), suggesting that OBP proteins possibly participate in the transport of exogenous substances in the hemolymph (Paskewitz and Shi, 2005; Armbruster et al., 2009). However, the precise functions of *OBP* genes in the insect fat body, Malpighian tubule, and hemolymph is unknown. Coincidentally, these tissues are involved in metabolic detoxification in insects (Beyenbach et al., 2010; Chen K. et al., 2019; Li et al., 2019). Therefore, we speculate that *TcOBPC11* in *T. castaneum* could play an important role in the degradation of exogenous substances.

OBPs in insects are frequently identified as proteins specifically involved in chemodetection. In recent years, functions of OBPs unrelated to chemodetection have been reported, including possible roles in morphology, egg and embryo development (Hassanali et al., 2005; Costa-da-Silva et al., 2013; Marinotti et al., 2014), delivering pheromones (Iovinella et al., 2011), avoiding cannibalism (Sun et al., 2012), regulating the melanization cascade (Benoit et al., 2017), and solubilizing nutrients (Zhu et al., 2016). The relationship between *OBPs* and insecticides in insects is also being investigated (Pelosi et al., 2018; Xiong et al., 2019). In *Plutella xylostella*, *OBP13* was strongly upregulated after treatment with permethrin (Bautista et al., 2015). In *Culex pipiens*, expression of *OBPjj7a* and *OBP28* in the 4th larval instar correlated positively with deltamethrin resistance (Li et al., 2016; Liu et al., 2017). Furthermore, *in vitro* binding tests found that OBPs have high binding affinity with insecticides (Li H. et al., 2015; Li et al., 2017; Zhang et al., 2020). Here, we investigated the OBP gene *TcOBPC11* in *T. castaneum* and revealed that expression of this gene was significantly induced by EO-AV. Moreover, RNAi of *TcOBPC11* directly correlated with an upgrade in the sensitivity of *T. castaneum* to EO-AV, providing evidence that *TcOBPC11* plays a crucial role in the defense of the red flour beetle against toxic substances. OBPs could act as binding proteins for exogenous toxicants in cellular detoxification systems in *T. castaneum* (Chen et al., 2016; Xiong et al., 2019). Knowledge on the mechanisms and functions of OBP genes in the defense of the red flour beetle against exogenous toxic substances could be applied to develop novel therapeutics against this serious agricultural pest.

## Data Availability Statement

The sequencing data has been deposited into GenBank (accession: XM_962706).

## Author Contributions

BL, YZ, and SG conceived and designed the experiments. SG and SX performed the experiments. YZ, JW, and KZ analyzed the data. YZ and BL wrote the manuscript. All authors contributed to the article and approved the submitted version.

## Conflict of Interest

The authors declare that the research was conducted in the absence of any commercial or financial relationships that could be construed as a potential conflict of interest.

## Supplementary Material

FIGURE S1 Phylogenetic tree of *T. castaneum* TcOBPC11 with homologous proteins from other insects. The phylogram was reconstructed using the neighbor-joining method in MEGA 6.1. Bootstrap values (2,000 replicates) are shown next to the branches. GenBank accession numbers and scientific names of insects are shown behind branches.

FIGURE S2 Relative expression of *TcOBPC11* and four non-target genes 36 h after injection of water or dsOBPC11. Control larvae were injected with the same amount of water. Asterisks and NS above the bars (mean ± SE, *n* = 3) represent the presence and absence of significant differences between injection of water and dsOBPC11 at the *P* < 0.05 level, respectively.
