# Supplementary figures and images for: Odorant-Binding Proteins Contribute to the Defense of the Red Flour Beetle, Tribolium castaneum, Against Essential Oil of Artemisia vulgaris
Source: Front Physiol. 2020 Aug 31;11:819. doi: 10.3389/fphys.2020.00819 (PMC7488584; doi:10.3389/fphys.2020.00819)

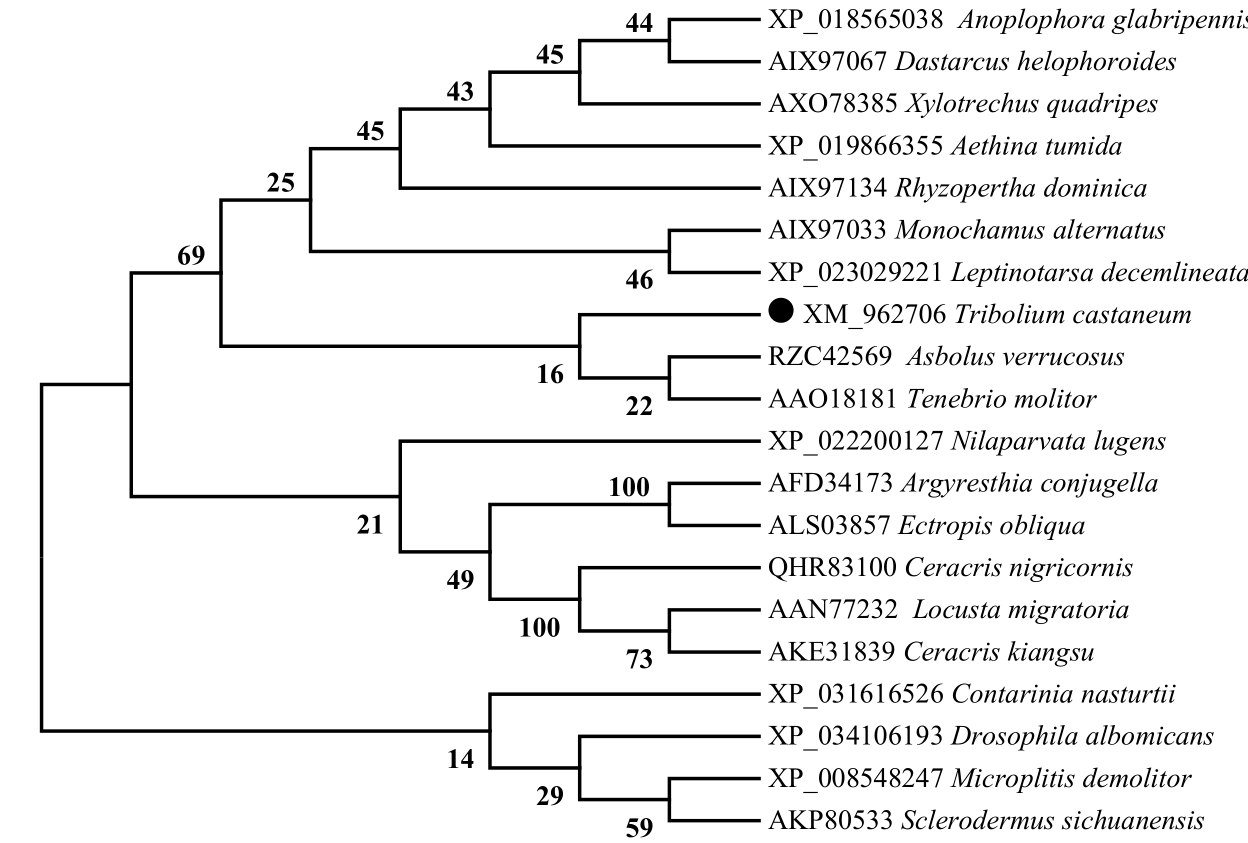

Supplement: FIGURE S1 — Phylogenetic tree of T. castaneum TcOBPC11 with homologous proteins from other insects. The phylogram was reconstructed using the neighbor-joining method in MEGA 6.1. Bootstrap values (2,000 replicates) are shown next to the branches. GenBank accession numbers and scientific names of insects are shown behind branches. [file Image_1.JPEG]

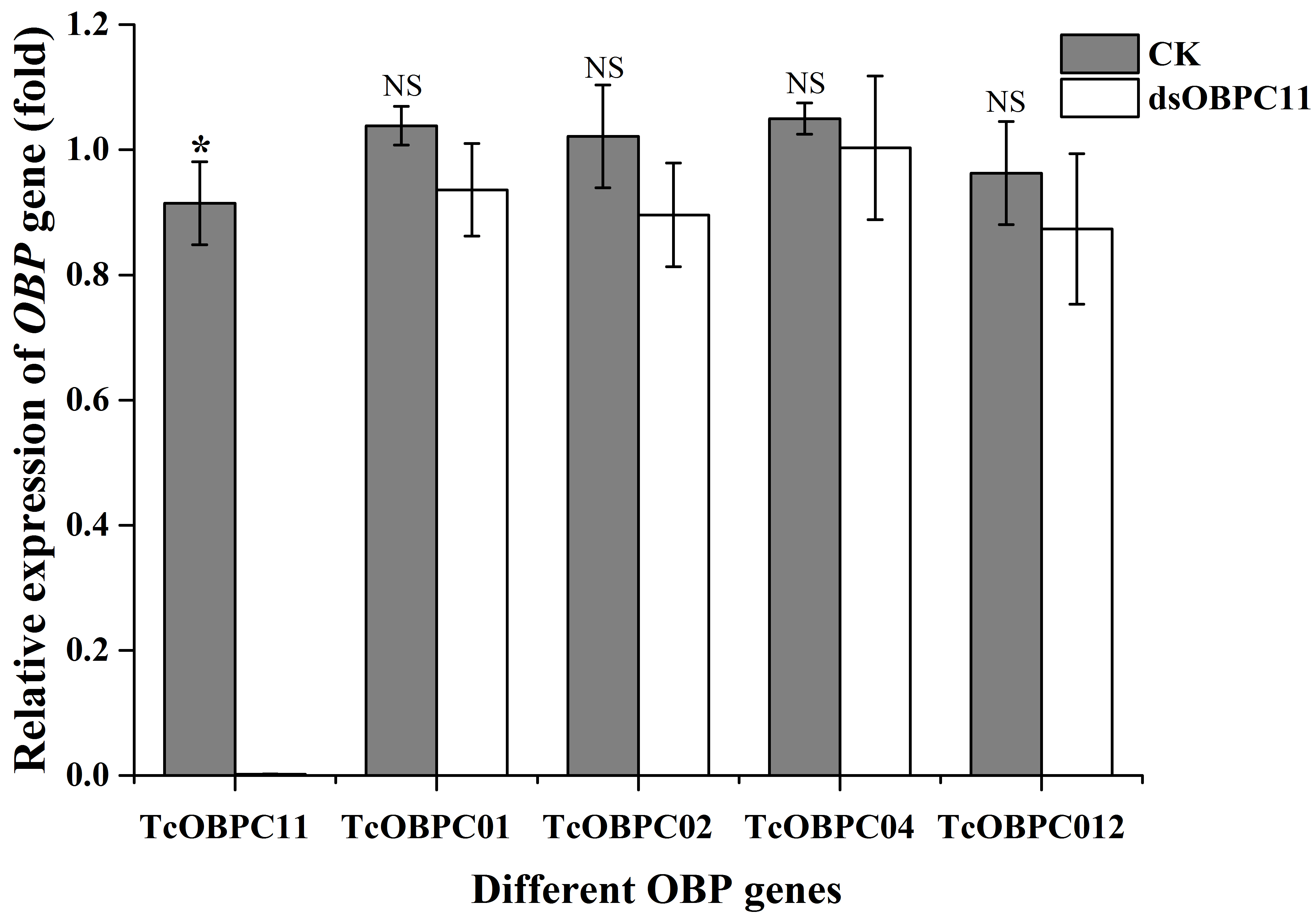

Supplement: FIGURE S2 — Relative expression of TcOBPC11 and four non-target genes 36 h after injection of water or dsOBPC11. Control larvae were injected with the same amount of water. Asterisks and NS above the bars (mean ± SE, n = 3) represent the presence and absence of significant differences between injection of water and dsOBPC11 at the P < 0.05 level, respectively. [file Image_2.JPEG]
