# Supplementary material for: Odorant-Binding Proteins Contribute to the Defense of the Red Flour Beetle, Tribolium castaneum, Against Essential Oil of Artemisia vulgaris
Source: Front Physiol. 2020 Aug 31;11:819. doi: 10.3389/fphys.2020.00819 (PMC7488584; doi:10.3389/fphys.2020.00819)
Supplement: Supplementary file 3 [file Table_1.docx]

**T**able **S1** Primers used for qRT-PCR analysis of non-target genes

| Primers | Sequences (5’-3’) | Product length | Remarks |
| --- | --- | --- | --- |
| OBPC01-F | AACCCAAACTGAAAAACCACG | 213 bp | qPCR |
| OBPC01-R | TGACCTTCCTCAAGCAAACGA |  |  |
| OBPC02-F | ACTAGAAACCCAATCACAATGAACTT | 108 bp | qPCR |
| OBPC02-R | AAGTTGGTTTAGCTTCTCAGTCTGTT |  |  |
| OBPC04-F | AAGAGTGGTAGTGGCTGTAGCG | 132 bp | qPCR |
| OBPC04-R | TTTTCTTTTTGCTCGTCAGTGAG |  |  |
| OBPC12-F | TCTTTGATCACAACGAAAATGAAA | 108 bp | qPCR |
| OBPC12-R | GTCGCTGTATTTCCTCAGCTTTT |  |  |

Note: F represents forward primers; R represents reverse primers.
