# Supplementary material for: Odorant-Binding Proteins Contribute to the Defense of the Red Flour Beetle, Tribolium castaneum, Against Essential Oil of Artemisia vulgaris
Source: Front Physiol. 2020 Aug 31;11:819. doi: 10.3389/fphys.2020.00819 (PMC7488584; doi:10.3389/fphys.2020.00819)
Supplement: Supplementary file 4 [file Table_2.docx]

**Table S2** List of chemosensory-related genes with at least 1-fold upregulation and a P-value less than 0.05 in 5% *A. vulgaris* treatment compared to control groups

| Gene family | Gene ID | Log_2_Ratio (TG/CK) | *P*-value | Corresponding gene |
| --- | --- | --- | --- | --- |
| Odorant binding protein | LOC656161 | 4.28 | 2.26E-65 | OBPC11 |
|  | LOC100240681 | 2.12 | 6.93E-09 | OBPC17 |
|  | LOC664595 | 1.91 | 2.23E-217 | OBP10 |
|  | LOC664599 | 1.41 | 5.71E-155 | OBPC02 |
|  | LOC656243 | 1.40 | 0 | OBPC12 |
|  | LOC664598 | -2.25 | 0 | OBPC01 |
| Chemosensory proteins | LOC661270 | 1.36 | 2.72E-31 | CSP12 |
|  | LOC657697 | 1.33 | 0 | CSP20 |
|  | LOC661469 | 1.24 | 0.000982 | CSP17 |
|  | LOC661219 | 1.12 | 1.22E-37 | CSP11 |
|  | LOC661799 | -2.15 | 9.46E-10 | CSP8 |
| Odorant receptor | LOC103312165 | 1.23 | 0.0001424 | OR4 |
|  | LOC107397828 | 1.75 | 0.0009787 | OR1-like |
|  | LOC100141826 | -1.30 | 7.83E-13 | OR22c |
|  | LOC107398416 | -6.32 | 5.63E-11 | OR45b |
|  | LOC107398667 | -1.55 | 0.0005917 | OR59c |
|  | LOC103314254 | -2.01 | 1.92E-14 | OR76 |
| Cytochrome P450 | LOC660270 | 4.03 | 0 | CYP4BN1 |
|  | LOC664471 | 2.04 | 5.36E-52 | CYP6BQ7 |
|  | LOC658048 | 2.03 | 2.33E-37 | CYP6A2 |
|  | LOC103313315 | 1.63 | 6.44E-23 | CYP351A2 |
|  | LOC664285 | 1.31 | 2.63E-37 | CYP9Z2 |
|  | LOC657454 | 1.27 | 2.98E-30 | CYP9AC1 |
|  | LOC656770 | 1.12 | 6.93E-55 | CYP6BK |
|  | LOC657560 | 1.07 | 3.78E-13 | CYP6A14 |
|  | LOC656306 | -1.22 | 3.25E-57 | CYP4BN5 |
|  | LOC659878 | -1.51 | 1.31E-71 | CYP4Q1 |
|  | LOC662300 | -1.99 | 3.03E-22 | CYP351A3 |
|  | LOC662337 | -2.49 | 6.97E-127 | CYP4C1 |
|  | LOC664475 | -2.87 | 5.91E-05 | CYP6A20 |
|  | LOC661930 | -3.09 | 5.08E-05 | CYP349A1 |
| Glutathione S-transferase | LOC657602 | 1.76 | 3.47E-76 | GST6 |
|  | LOC659009 | 1.13 | 4.50E-45 | GST7 |
|  | LOC655331 | -1.24 | 5.47E-242 | GST7 |
|  | LOC655415 | -1.33 | 3.03E-92 | GST 8 |
|  | LOC107398495 | -2.77 | 7.86E-25 | GST4 like |
| Esterase | LOC662506 | 1.87 | 0 | EST5 |
|  | LOC661102 | 1.43 | 1.12E-09 | EST2 |
|  | LOC661621 | 1.37 | 0 | EST6 |
